# Magnetic Resonance Imaging and Bioluminescence Imaging for Evaluating Tumor Burden in Orthotopic Colon Cancer

**DOI:** 10.1038/s41598-019-42230-w

**Published:** 2019-04-15

**Authors:** M. K. Ravoori, O. Margalit, S. Singh, Sun-Hee Kim, W. Wei, D. G. Menter, R. N. DuBois, V. Kundra

**Affiliations:** 1 Department of Cancer Systems Imaging, The University of Texas MD Anderson Cancer Center, 1400 Pressler St., Houston, TX 77030 USA; 2 Department of Oncology, Chaim Sheba Medical Center, Sackler School of Medicine, Tel-Aviv University, Tel-HaShomer, 52621 Israel; 3 Department of Melanoma Medical Oncology, The University of Texas MD Anderson Cancer Center, 1400 Pressler St., Houston, TX 77030 USA; 4 Department of Biostatistics, U.T.-M.D. Anderson Cancer Center, 1400 Pressler St., Houston, TX 77030 USA; 5 Department of Gastrointestinal Medical Oncology, Division of Cancer Medicine, U.T.-M.D. Anderson Cancer Center, 1515 Holcombe Blvd., Houston, TX 77030 USA; 6 MUSC College of Medicine, Dean’s Office, 96 Jonathan Lucas Street, Suite 601, MSC 617, Charleston, SC 29425 USA; 7 Department of Radiology, U.T.-M.D. Anderson Cancer Center, 1400 Pressler St., Houston, TX 77030 USA

## Abstract

Quantifying tumor burden is important for following the natural history of orthotopic colon cancer and therapeutic efficacy. Bioluminescence imaging (BLI) is commonly used for such assessment and has both advantages and limitations. We compared BLI and magnetic resonance imaging (MRI) for quantifying orthotopic tumors in a mouse model of colon cancer. Among sequences tested, T2-based MRI imaging ranked best overall for colon cancer border delineation, contrast, and conspicuity. Longitudinal MRI detected tumor outside the colon, indistinguished by BLI. Colon tumor weights calculated from MRI *in vivo* correlated highly with tumor weights measured *ex vivo* whereas the BLI signal intensities correlated relatively poorly and this difference in correlations was highly significant. This suggests that MRI may more accurately assess tumor burden in longitudinal monitoring of orthotopic colon cancer in this model as well as in other models.

## Introduction

Animal models, specifically murine models, are widely used in cancer research and contribute critically to expanding our knowledge of cancer biology and assessing the efficacy of potential treatments before performing clinical trials. Tumor development is affected by many factors, including interactions between cancer cells and their environment^1–3^; thus, orthotopic locations are preferred among tumor models^4–7^.

To gain a proper understanding of tumor development and the effects of interventions, longitudinal analyses are desirable^8^. Unlike subcutaneous tumors, most orthotopic tumors, such as colon cancer, are not amenable to measurement by calipers^6,8^ and colon tumors, particularly in the cecum, are commonly difficult to reach for direct observation by mouse colonoscopy. Instead, non-invasive imaging is needed. Light-based methods such as BLI require gene insertion; thus, they are useful in most cell lines, but not in xenograft models, and may affect the immunogenicity of the modified cells^9^. MRI does not require gene transfer and is useful for both cell lines and xenograft models^8,10–12^. BLI has been promulgated as a method of quantifying tumor load in animals and is reasonably valid when performed in a standardized manner^6,9^; it is also relatively rapid, inexpensive, and easy to perform. It has been applied to multiple tumor types. Signal can be influenced by various factors such as pH and hypoxia and signal can be attenuated by tissue and hair^13–16^.

The utility of MRI has been demonstrated in different types of orthotopic tumors^8,11,17^. MRI has high spatial resolution, does not require gene transfer into cells or radioactivity, and is generally not limited by tissue depth; in addition, hair does not interfere with the signal. Because multiple different sequences can be applied, selecting the appropriate sequences for depicting the tumor is important. The present study relied only on endogenous MRI tissue signal and did not use exogenous contrast agents.

For MRI, various sequences may be applied, however, these have not been compared for which best depicts orthotopic colon cancer. Further, whether MRI or BLI better depict orthotopic colon cancer needs clarification. We compared two of the most widely used imaging modalities, MRI and BLI, for monitoring tumor progression in an orthotopic mouse model of colon cancer.

## Results

**Figure 1:**
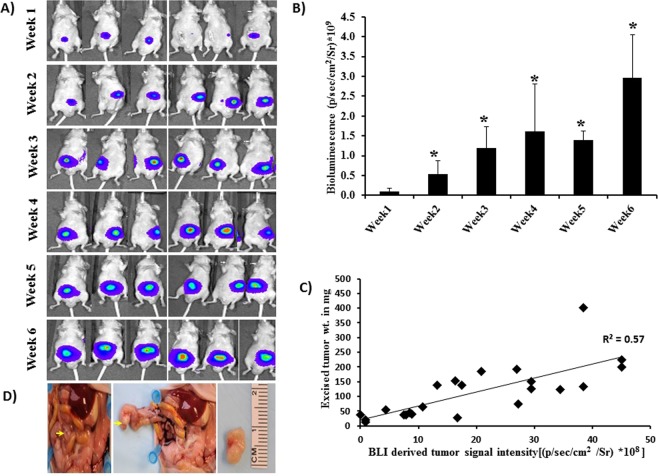
Monitoring colon tumors by BLI. (**A**) Longitudinal BLI of human HCT-116-Luc derived tumors in athymic nude mice. (**B**) BLI radiant efficiency versus time. Increase in BLI total signal intensity over time suggests progression of cancer (*compared to week1, n = 6, p ≤ 0.05). (**C**) Correlation between *in vivo* radiant efficiency (photons/sec/cm^2^/Sr) and excised tumor weight (*r*^*2*^ = 0.55, *n* = 24, p ≤ 0.001). (**D**) Representative example of primary colon tumor at necropsy and size of the tumor 6 weeks after cell inoculation in the colon. Arrow, tumor.

**Figure 2:**
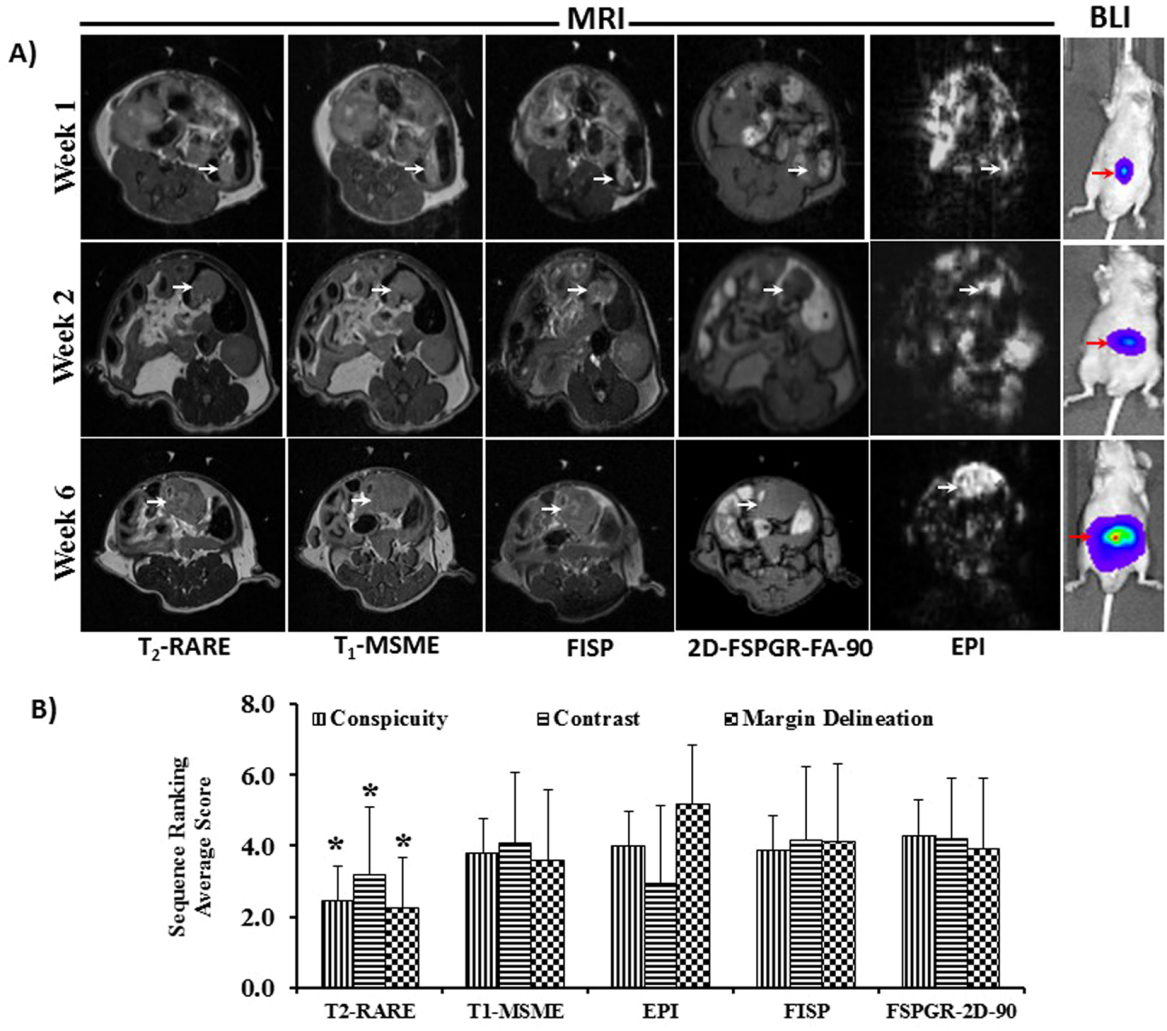
Monitoring colon tumors by MRI. (**A**) Representative axial *in vivo* MRI and coronal *in vivo* BLI longitudinal images of the same mouse colon HCT-116-Luc tumor. (**B**) Colon tumor conspicuity, contrast, and margin delineation of athymic nude mice overall ranked significantly higher on T2-weighted MRI (*p ≤ 0.001, n = 24, T2-weighted RARE vs all other sequences).

**Figure 3:**
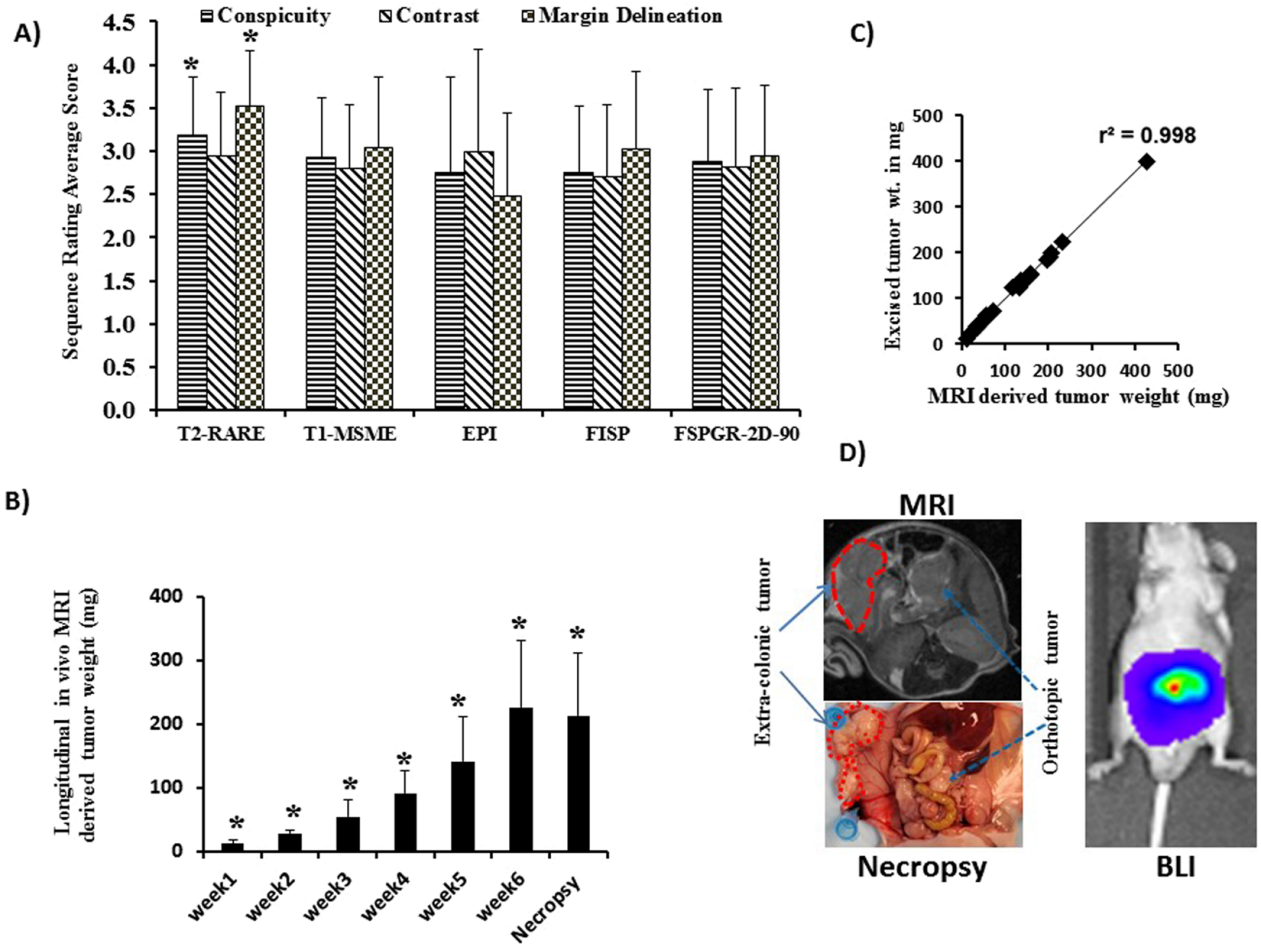
Quantitative measurements of colon tumors by MRI. (**A**) Colon tumor conspicuity and margin delineation rated significantly higher on T2-weighted MRI (*p ≤ 0.001, n = 24). (**B**) MRI derived tumor weight versus time. Increase in MR-derived tumor weight suggests progression of cancer (*compared to week1, n = 6, p ≤ 0.05). (**C**) Tumor weights determined by MRI correlated with tumor weights of excised tumors (*r*^*2*^ = 0.998, *n* = 24). (**D**) Example of extracolonic and colonic tumors in the same mouse 5 weeks after HCT-116-Luc cell implantation in the colon detected by axial T2-weighted MRI (left top) and confirmed at necropsy (left bottom). The two tumors were not distinguished by BLI (right).

Tumor take rate was 100% with HCT-116-Luc cells, with tumor formation in all 24 animals implanted. One week after cell inoculation, BLI and MRI were performed. Tumor growth at the primary site was evident by BLI and MRI as early as 1 week after cell inoculation (Figs 1A and 2A). Figure 1A shows BLI of animals longitudinally over a period of 6 weeks; and, Fig. 2A shows representative longitudinal MR images using different sequences of a tumor from the same mouse. Note that the T2-weighted images separate the tumor from the surrounding high-signal-intensity fat and low-signal-intensity air; this sequence ranked the highest among the sequences presented per mouse (*n* = 24, *p* ≤ 0.001) for colon tumor conspicuity, contrast, and margin delineation (Fig. 2B) and when sequences were presented randomly rated best for overall tumor conspicuity and margin delineation (Fig. 3A). Thus, the T2-based sequence was used to derive the tumor weight.

**Figure 4:**
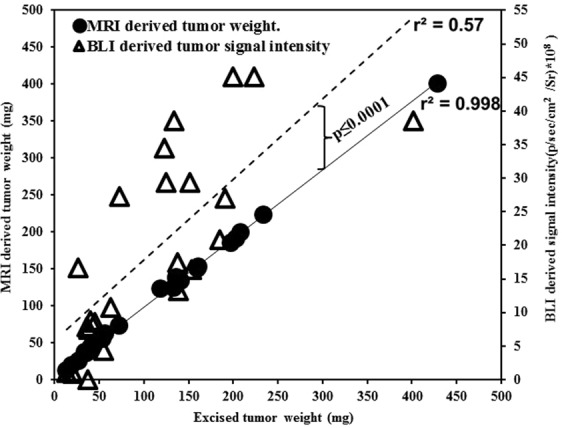
Comparison of correlation of tumor burden by MRI and BLI with *ex vivo*. Correlation of *ex vivo* tumor weight with the *in vivo* MRI-derived weight (r^2^ = 0.998, n = 24, solid line) and of *ex vivo* tumor weight with BLI-derived tumor radiant efficiency (*r*^*2*^ = 0.57, *n* = 24, dashed line). The correlations were statistically significantly different (*p* < 0.0001, *n* = 24).

Quantification of the BLI signal and MRI-derived tumor weight over time demonstrated progressive increase in tumor growth **(**Figs 1B and 3B). The BLI signal intensities correlated with tumor weights measured *ex vivo* (*r*^2^ = 0.57, *n* = 24, *p* ≤ 0.001; Fig. 1C), but the colon tumor weights calculated from MRI *in vivo* and the weights measured *ex vivo* were highly correlated with each other (*r*^2^ = 0.998, *n* = 24, *p* ≤ 0.001; Fig. 3C3). Tumors outside the colon were observed in 3 of 24 animals that underwent MRI (Fig. 3D), suggesting metastatic disease versus seeding from the initial implantation. Tumors within versus outside the colon were not differentiated by BLI (Fig. 3D). The *ex vivo* tumor weight correlation with MRI-derived tumor weight was similar when mice with tumors outside the colon were excluded (*r*^2^ = 0.996, *n* = 21). The correlation between BLI signal intensity and MRI-derived tumor weight was similar, whether including the animals with tumor outside the colon (*r*^2^ = 0.57, *n* = 24) or excluding them (*r*^2^ = 0.58, *n* = 21). Also, the correlation between *ex vivo* tumor weight with BLI signal intensity was similar, whether including the mice with tumor outside the colon (*r*^2^ = 0.57, *n* = 24) or excluding them (*r*^2^ = 0.58, *n* = 21). The Steiger method^18^ was used to compare the data in Figs 1C and 3C, i.e. the correlation of the *ex vivo* tumor weight with the *in vivo* BLI-derived tumor signal intensity versus *ex vivo* tumor weight with the *in vivo* MRI-derived weight; these were statistically significantly different (*p* < 0.001, *n* = 24, Fig. 4), indicating that the MRI-derived tumor weight is superior to BLI-measured signal intensity values for estimating tumor burden.

**Figure 5:**
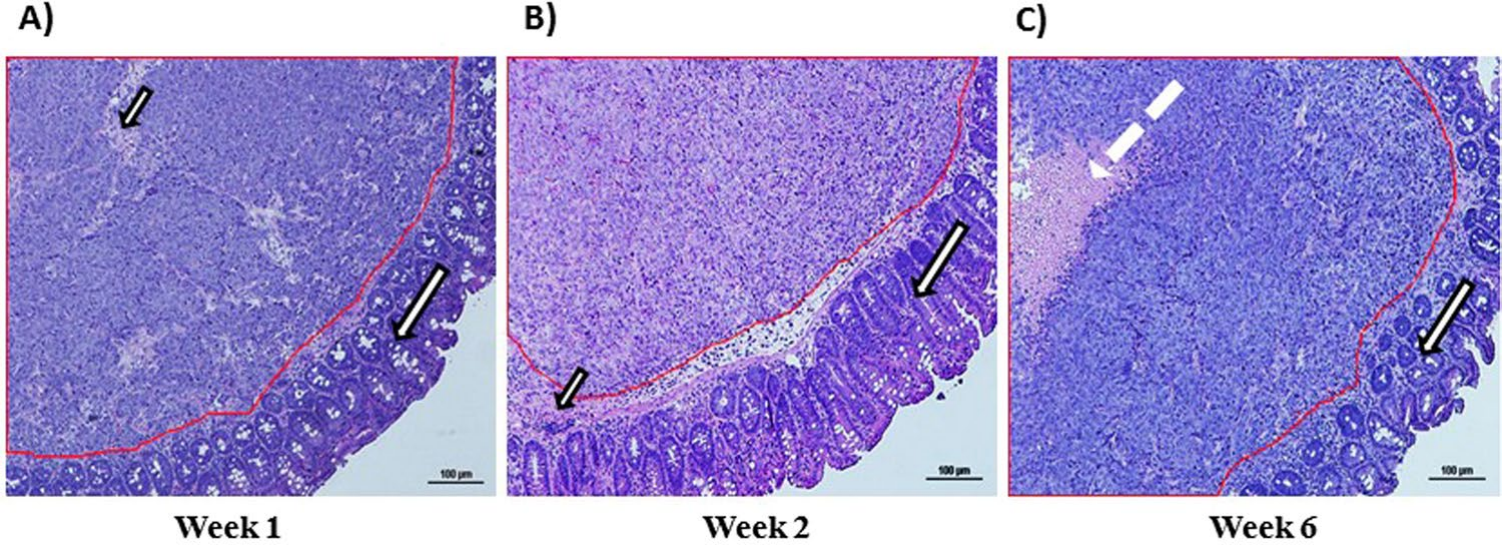
Histology of orthotopic colon tumors. (**A**) One week after tumor cell implantation in the colon; (**B**) 2 weeks after tumor cell implantation in the colon; and (**C**) 6 weeks after tumor cell implantation in the colon. Magnification, 4X. Long arrow: normal mucosa, Red outline area: tumor beneath the mucosa, dashed arrow: necrosis within the tumor, and short arrow: connective tissue.

Histopathological examination revealed clusters of large cells with a large oval or round acidophilic cytoplasm in well-differentiated tumors (Fig. 5A–C). Histological examinations of tumor sections from mice euthanized at week 6 revealed small areas of hemorrhage or infarction within the tumor (Fig. 5C).

## Discussion

T2-weighted MR imaging better depicted orthotopic colon tumors compared to BLI in the mouse model, enabling longitudinal tumor monitoring *in vivo*. Rating and ranking studies found the T2-weighted sequence to be superior to other MR sequences tested. The findings were supported by the high correlation between *in vivo* MRI-derived tumor weights and *ex vivo* tumor weights^19^. In comparison, BLI signal intensities correlated less well with *ex vivo* colon tumor weights. Moreover, unlike BLI, MRI distinguished colonic vs extracolonic tumors. Longitudinal BLI and MRI confirmed the progression of orthotopic tumor growth with time. As far as we are aware, multiple MRI sequences for imaging colon tumors have not been compared in orthotopic mouse models. In the current study, we found that among the MRI sequences, the T2-weighted sequence fared best for lesion detection and correlated highly with the excised tumor weight. These findings were supported by reader studies in which T2-based sequences ranked higher than other sequences for colon tumor conspicuity, contrast, and margin delineation. Intraperitoneal tumor can be seen with cancer metastasis in humans. Two-dimensional BLI, used as a semiquantitative tool to measure tumor burden^9,20^, did not distinguish between tumors inside and outside the colon. In addition, BLI tumor signal intensity is dependent on several factors such as the lesion depth and is limited by its need for tumors to express a luciferase reporter gene^9,13–16,20^; thus, BLI is possible in cultured cell tumor models but can be significantly more challenging in spontaneous, transgenic, or xenograft models. In contrast to optical imaging techniques, it should be possible to perform MRI in most colon tumor-bearing mice, including spontaneous, transgenic and xenograft models, such as patient-derived xenografts.

Studies comparing BLI and MRI to gold standard excised tumor weight are wanting. Jost *et al*.^21^ compared BLI and MRI derived tumor volumes in a mouse model of glioblastoma and found a moderate correlation (*r*^2^ = 0.78) among smaller tumors (less than 40 mm^3^ volume) and a comparative loss of this correlation (*r*^2^ = 0.13) with larger tumors that grew beyond 40 mm^3^; they then utilized both BLI and MRI together to measure tumor volumes for their preclinical evaluations; the study compared BLI to MRI measured tumor volumes but did not compare to gold standard excised tumor weights/volumes. In a hepatocellular carcinoma model, Wu *et al*.^22^ found *r*^2^ = 0.43 comparing BLI to tumor size by MRI. Thalhemier *et al*.^23^ found *r*^2^ = 0.83 comparing BLI vs number of colon cancer (HCT-116) liver metastases, Ramasawmy *et al*. found *r*^2^ = 0.98 comparing BLI to MRI tumor burden ((tumor volume/liver volume) × 100) in a SW1222 colon liver metastasis model^24^ whereas Fleten *et al*.^25^ found *r*^2^ = 0.55 comparing BLI of HCT116 colon liver metastases to excised tumor weight.

The ability to visualize and quantify tumor burden before therapy aids evaluation of treatment response. The present study demonstrated that to measure tumor growth longitudinally, T2-weighted MRI better depicted the orthotopic colon tumors than did the 2D BLI. Currently, nearly all BLI as practiced is performed in 2D. In the current study, 2D analysis did not distinguish colonic from extracolonic tumor but instead merged them into one global signal. Recently, bioluminescence imaging system have been equipped with software specifically designed to run 3D Diffuse Luminescent Imaging Tomography (DLIT) algorithms^26,27^. Whether these will aid distinguishing colonic vs extracolonic tumors or demonstrate improved correlation with tumor burden needs to be tested.

Relative merits of BLI and MR imaging are presented in Table 1. For 48 mice, the cost of MR may be approximately ten fold greater than BLI depending on institutional cost structure and number of mice imaged at a time. The balance of experiment cost vs time and image sensitivity need to be closely examined for every study. Given cost and time, BLI may be appropriate for several applications, particularly when alterations in tumor burden are expected to be large. It may also be appropriate for detecting smaller, live tumors expressing luciferase that may not be detectable by MR. We found that MR has the advantage of higher correlation with tumor burden than BLI, which may be needed in several applications such as assessing response to therapy. Approximately 0.75 correlation between BLI and MR have been found in other models such as glioma^28^. In the current study, compared to *ex vivo* tumor burden assessment, BLI correlation coefficient was approximately 0.75, whereas, MR was 0.99. MR also may be useful in transplantable models and in established genetically modified animal models where gene transfer of luciferase is difficult. In some situations, BLI and MR may be complimentary, such as using the former for earlier detection of tumors expressing luciferase and the latter for assessing the primary and metastases. Other techniques may also have advantages such as high-frequency US for tumor burden assessment^29^ and functional assessment, and may be further compared in the future.

**Table 1:** Relative advantages and disadvantages of the BLI and MRI techniques.

Imaging Modality	Imaging Resolution/level of difficulty	8 hour day Imaging Capabilities	Clinically Translatable	Estimated Material & Imaging Cost/hour^a^	Imaging time (minutes)
**Magnetic Resonance Imaging (MRI)**	**Pros**-higher resolution of ~200 µm in plane and accurately assess location and the tumor burden within colon. - easier to identify metastatic disease versus primary tumor. -3D imaging, can provide accurate quantitative data & response to therapy. -No contrast injection needed, can differentiate endogenous tissue contrast - does not require transfection of cells or radioactivity, and hair on the skin does not interfere with the signal. **Cons-** limited detection of tumor growth early after implantation. May be less sensitive for minute tumors (sub 150 micron tumors) depending on image resolution. - may be relatively expensive, somewhat labor intensive - requires higher technical expertise that may be obtained from core facilities.	~48 mice^b^	Yes	$160	**Core sequences:** ~ **7** min. Scan + **3–5** min. animal set up^b^ i.e. Scout- **2** min. Tripilot-**2** min. T_2_-RARE-**3** min. Other sequences used in this study: T1-MSME- **3** min. FISP- **2** min. 2D-FSPGR-**2** min. EPI- **30** sec. **Generally:1 mouse per scan** ^b^
**Bioluminescence Imaging (BLI)**	**Pros-** detect small number of living tumor cells. Detection is variable and depends on degree of luciferase expression. - ease of image acquisition, rapid, high throughput, and relatively low cost of operation. -can be performed with basic training in the technique & computer skills requiring less technical expertise. **Cons-**2D imaging, provides semi-quantitative data. -exact location and depth can’t be measured precisely, which can limit evaluation of metastases vs primary - hair on the skin can interfere with the signal. - Cells have to be genetically modified by luciferase limiting applicability to some transplantable and genetically modified mouse models.	~288 mice	No	$50–75	~5–30 min, wait between injection & imaging. ^c^ **30–60** seconds scan + 4 min animal set up **Generally: 3 mice per scan**

^a^Cost will vary by institution. We used in house charges at our institution for the example. ^b^Scan time can be reduced per mouse if multiple animals are imaged at once. ^c^Depends on injection route.

In the present study, orthotopic tumor growth was readily detected and observed over time by serial BLI and MRI measurements. BLI is a sensitive semi-quantitative tool for longitudinally monitoring orthotopic colon cancer. MRI can longitudinally detect orthotopic colon cancer and more accurately assess tumor burden. MRI should provide a safe, non-invasive method for monitoring the effects of new therapeutics, determining the optimal time for the collection of samples for molecular analysis, and correlating growth response with other functional response indicators. Colonoscopy is the primary screening test for colon cancer in patients. Radiologically, colon tumor detection has relied on CT colonography, which is commonly used in circumstances such as failed or incomplete colonoscopy or patient preference. MR is already used clinically for staging of rectal tumors. The current study suggests potential for clinical translation of MRI in the setting of colon tumors.

## Conclusion

Among MR sequences tested, the T2-weighted sequence best depicted orthotopic colon tumors. BLI may have sufficient accuracy for assessing tumor burden in several scenarios evaluating orthotopic colon cancer and has advantages of low cost and ease of use. MRI better depicted the orthotopic colon tumor location and volumes/weights than did BLI; thus, MRI may more accurately assess tumor burden in longitudinal monitoring of orthotopic colon cancer in this model as well as in other models, which may be advantageous in a variety of applications.

## Materials and Methods

### Animals

All mice were housed and treated in accordance with protocols approved by the Institutional Animal Care and Use Committee at The University of Texas MD Anderson Cancer Center (Houston, Texas). 24 (8-to-9-week-old) male athymic nude mice (*nu/nu*) (body weight, 20–25 g) were purchased from Harlan Sprague Dawley, Inc. (Indianapolis, IN, USA).

### Cancer cell lines

Human colon cancer HCT-116 cells were purchased from the American Type Culture Collection. Cells were maintained in McCoy 5A medium containing 10% fetal bovine serum in a 5% CO_2_ atmosphere.

### Establishment of HCT-116-Luc cells

293T cells were infected with a lentiviral vector carrying a firefly luciferase gene, along with package vectors psPAX2 and pMD2.G, in 60-mm dishes using Lipofectamine 2000 reagent (Invitrogen, Carlsbad, CA), according to the manufacturer’s protocol. Culture medium containing virus particles was collected 48 hours later and added to the HCT-116 cells, which were then selected with 1 μg/ml puromycin to eliminate uninfected cells.

### Orthotopic animal model

For the orthotopic xenograft mouse model, 1 × 10^5^ luciferase-expressing HCT-116 cells (HCT-116-Luc) were suspended in 30 μl of HBSS medium and injected into the cecal wall of 24 8-9-week-old male athymic nude mice, as previously described^3^. To minimize the retrograde leakage of cells into the peritoneum, a 1.5-mm diameter fat pad was harvested from the abdominal fat of a donor male nude mouse and was adhered using surgical glue (Dermabond; Ethicon, Inc., Somerville, NJ) on top of the injection site. If any fluid seepage occurred from the injection site, the mouse was excluded from the study to limit extracolonic tumor related peritoneal tumor formation. For tumor monitoring, groups of 3 mice were imaged then sacrificed each week to analyze the distribution and weight of tumors for a period of 6 weeks; another 6 mice were imaged longitudinally for 6 weeks and then euthanized to analyze tumor distribution and weight.

### BLI

For BLI, mice were injected intraperitoneally with 150 mg/kg of D-Luciferin (Caliper Life Sciences). After 10 minutes, mice were anesthetized with 2% isoflurane; they were then placed supine, with their abdomen toward the camera, and imaged, following the manufacturer’s recommendations, for 10 seconds using the Xenogen IVIS 200 BLI system (Caliper Life Sciences). Using Living Image 2.5 software, regions of interest (ROIs) were drawn around the tumor and total radiant efficiency (in photons/sec/cm^2^/Sr) of the tumor was measured.

### MRI

For MRI, animals were anesthetized with 2% isoflurane. Body temperature was maintained at 37 °C by laying the mice on a bed heated with circulating warm water. The animals were imaged in a prone position. MRI data were collected on a Bruker MR (Bruker Biospec, Bruker Biospin, Billerica, MA) with a 7T/200-mm horizontal bore magnet and a 150 milli-Tesla/meter (mT/m) gradient insert. After scout images were obtained, the following imaging parameters were used to acquire the images: T2-weighted rapid acquisition with relaxation acquisition (echo time, 38 ms; echo train length, 8; receiver bandwidth, 101 kHz; repetition time, 2453.85 ms; nex, 4; slice thickness, 0.75 mm; field of view, 4 × 3 cm; matrix, 256 × 192; flip angle, 180°), T1 multi-slice multi-echo (echo time, 20 ms; repetition time, 905.25 ms; nex, 1; slice thickness, 0.75 mm; field of view, 4 × 3 cm; matrix, 256 × 192; spatial resolution, 156 µm), echo planar imaging (echo time, 24.67 ms; echo train length, 1; repetition time, 1684.50 ms; nex, 6; slice thickness, 0.75 mm; field of view, 4 × 3 cm; flip angle 90°), fast imaging steady precession (echo time, 2 ms; echo train length, 128; repetition time, 4 ms; nex, 4; slice thickness, 0.75 mm; field of view, 4 × 3 cm; flip angle, 60°), and two-dimensional fast spoiled gradient-recalled echo (echo time, 1.3 ms; echo train length, 1; repetition time, 123.08 ms; nex, 4; slice thickness, 0.75 mm; field of view, 4 × 3 cm; matrix, 128 × 96; flip angle, 60°, 75°, and 90°).

### Interpretation of MRI

To rate the quality of colon tumor border delineation, contrast, and conspicuity, all MRI sequence images were presented randomly; blinded interpretation was performed at different times by a board-certified radiologist and a research scientist focused on small animal imaging. The images were rated on a scale of 1 to 5, with 1 = not seen, 2 = poor, 3 = fair, 4 = good, and 5 = excellent. In addition, the different sequence-derived images for each mouse were presented and ranked in order from 1 to 7, with 1 being the best.

### Colon tumor measurements on MRI

Multiple 0.75-mm slices that spanned the entire colon were used to generate the ROIs. These were manually traced around tumors in each slice using the National Institutes of Health’s ImageJ software^27^. The sum of the volumes of contiguous tumor ROIs was tabulated; to control for volume averaging, ½ the volume of the most cranial or caudal image was added. Assuming a tumor density of 1 g/ml, tumor volumes (mm^3^) were converted to weight (g) for analysis.

### Histological examination

Colon tumors were fixed with 4% formaldehyde, dehydrated in ethanol, and embedded in paraffin. Five-micrometer-thick sections were cut, dried, mounted on glass slides, deparaffinized in xylene, and rehydrated in a series of ethanol and phosphate-buffered saline^28^. Sections were stained using hematoxylin and eosin.

### Statistics

Pearson correlation coefficients between BLI tumor signal intensity, MRI-derived tumor weight, and gold standard *ex vivo* tumor weight as well as t-tests were performed using spreadsheet software (Microsoft Office Excel 2003, Microsoft, Seattle, WA). For comparing rating and ranking scores for MR sequences, linear mixed model was performed using SAS version 9 (SAS Institute, Cary, NC). Reader, time, and mice were treated as random effects to account for correlations between readings from the same mice. Pairwise comparisons were made between all pairs of sequences. Tukey-Kramer adjustment was used to control overall type I error rate at 5%. Differences in the correlation between *ex vivo* tumor weight and BLI signal intensity versus *ex vivo* tumor weight and MRI-derived tumor weight were evaluated using the Steiger method^18^ and for this, the signal intensity was square root transformed while the MRI-derived tumor weight and true weight were log-transformed. All tests were 2-sided, and *p*-values of 0.05 or less were considered statistically significant.
